# Dysfunction of Telomeric Cdc13-Stn1-Ten1 Simultaneously Activates DNA Damage and Spindle Checkpoints

**DOI:** 10.3390/cells13191605

**Published:** 2024-09-25

**Authors:** Nathalie Grandin, Michel Charbonneau

**Affiliations:** GReD Institute, CNRS UMR6293, INSERM U1103, Faculty of Medicine, University Clermont-Auvergne, 28 Place Henri Dunant, BP 38, 63001 Clermont-Ferrand Cedex, France; nathalie.grandin@uca.fr

**Keywords:** budding yeast telomeres, Cdc13-Stn1-Ten1 complex, Siz1 SUMO E3 ligase, cell cycle, Bub2 and Mad2 spindle checkpoints, Mec1 DNA damage checkpoint

## Abstract

Telomeres, the ends of eukaryotic linear chromosomes, are composed of repeated DNA sequences and specialized proteins, with the conserved telomeric Cdc13/CTC1-Stn1-Ten1 (CST) complex providing chromosome stability via telomere end protection and the regulation of telomerase accessibility. In this study, *SIZ1*, coding for a SUMO E3 ligase, and *TOP2* (a SUMO target for Siz1 and Siz2) were isolated as extragenic suppressors of *Saccharomyces cerevisiae* CST temperature-sensitive mutants. *ten1*-*sz*, *stn1*-*sz* and *cdc13*-*sz* mutants were isolated next due to being sensitive to intracellular Siz1 dosage. In parallel, strong negative genetic interactions between mutants of CST and septins were identified, with septins being noticeably sumoylated through the action of Siz1. The temperature-sensitive arrest in these new mutants of CST was dependent on the G2/M Mad2-mediated and Bub2-mediated spindle checkpoints as well as on the G2/M Mec1-mediated DNA damage checkpoint. Our data suggest the existence of yet unknown functions of the telomeric Cdc13-Stn1-Ten1 complex associated with mitotic spindle positioning and/or assembly that could be further elucidated by studying these new *ten1*-*sz*, *stn1*-*sz* and *cdc13*-*sz* mutants.

## 1. Introduction

Telomeres are composed of TG-rich DNA sequences and specialized binding proteins, together forming high-order assemblies. They protect the ends of the linear chromosomes from being recognized as DNA double-strand breaks by DNA damage signaling and repair machineries [1]. In dividing cells, telomeres shorten at each cell division due to incomplete lagging-strand DNA replication, oxidative damage and exonucleolytic processes [2]. Excessive telomere erosion elicits DNA damage that activates cell cycle checkpoints, leading to senescence or apoptosis. The action of telomerase, a specialized reverse transcriptase [3,4], at telomere ends or the activation of the alternative lengthening of telomeres pathway (ALT) [5] compensate for this erosion of telomeres, notably in cancer cells. The existence of numerous telomere-impacting genetic disorders implicated in cancer, aging and other diseases [6,7,8] have contributed to the importance of telomere biology research over the last three decades.

In vertebrates, telomere protection is provided mainly by shelterin, a complex of six proteins (TRF1, TRF2, POT1, TIN2, TPP1 and RAP1) that prevents inappropriate recombination and fusion between telomeres. Shelterin subunits also have complementary roles in telomere replication and length regulation [9,10,11]. A similar complex exists in the fission yeast *Schizosaccharomyces pombe* [12], while a somewhat simpler protection complex, consisting mainly of the Cdc13, Stn1 and Ten1 proteins (the CST complex) is present in the budding yeast *Saccharomyces cerevisiae* [13,14]. *S*. *cerevisiae* CST is a telomere-specific Replication Protein A-like complex that plays a central role in telomere homeostasis and chromosomal end protection through its telomere capping and telomerase activation and inhibition functions [15,16,17,18,19]. Strikingly, orthologs of the three *S*. *cerevisiae* CST proteins are found in humans and mice, as well as in the plant *Arabidopsis thaliana* [20,21]. In *S*. *pombe*, Stn1 and Ten1, but not Cdc13/CTC1, have been identified as CST subunits [22].

Human and mouse CST genes were initially discovered by virtue of homology of their STN1 subunit to *S*. *cerevisiae* Stn1, followed by spectrometry analyses to identify the CTC1 and TEN1 subunits [20]. Human CST limits telomerase access at telomeres [23] and associates with shieldin at damaged telomeres, regulating, in association with Polα, the fill-in of the resected overhangs to facilitate DNA repair [24]. Importantly, hCST subunits were found via immunofluorescence to localize at only a small fraction of telomeres, and the hypersensitivity of mutants of hCST to DNA-damaging agents indicated a function in ensuring a genome-wide replication restart in response to DNA replication stress and replication fork stalling [20,25,26,27]. Finally, hCST was found to have a role in the initiation of DNA replication in association with MCM proteins [28] and also to physically associate with the cohesin complex, perhaps to prevent premature cohesion loss between chromosomes at stalled replication forks [29]. In addition, *A*. *thaliana* TEN1 was recently shown to act as a heat-shock-induced molecular chaperone, potentially protecting CTC1 from forming aggregates [30].

Given the conservation of CST during evolution, a complete understanding of its functions is needed now more than ever before [10,11,27,31]. In *S*. *cerevisiae* CST, Cdc13 has the most important role due to the high specificity of its single-stranded DNA DNA-binding capacity, ensuring telomere replication by recruiting telomerase and telomere end protection by associating with Stn1 [15,32,33]. Stn1 is essential for telomere end protection and also functions in telomere length regulation [17], being responsible, in association with DNA Polα, for the fill-in of the strand previously elongated by telomerase [34,35]. Ten1, however, has no known specific telomeric function besides being, like Cdc13 and Stn1, essential for telomere end protection and telomere length regulation [18]. Ten1 also acts to improve Cdc13 stability at the telomeres via direct binding [36]. In addition, *S*. *cerevisisae* Cdc13-Stn1-Ten1 might have another (extratelomeric) function in regulating transcription elongation [37], a function recently proposed to be defective in human colorectal cancers [38].

In this study, we set out to develop a novel strategy to uncover novel loss-of-function mutants of CST, and to do this, a temperature-sensitive synthetic *CDC13*-*TEN1*-*STN1* fusion gene, called *CST1*, was expressed in a strain in which the three corresponding (essential) genes had been deleted. Genetic suppressor screens allowed us to isolate *SIZ1*, coding for a SUMO E3 ligase, and *TOP2*, coding for topoisomerase II, as extragenic suppressors of these mutants. In parallel, we identified strong negative genetic interactions between the mutants of CST and septins; notably, septins are sumoylated through the action of Siz1. Septins are GTP-binding, cytoskeletal proteins conserved from yeast to humans, having been first identified in budding yeast for their essential role in cytokinesis and also for playing critical roles in diverse functions including morphogenesis, mitosis, cell migration, ciliogenesis and exocytosis [39,40]. Septins form heterooligomeric complexes polymerizing end-to-end into filaments that can be further organized into higher-order structures, such as rings and hourglasses, depending on cell types or cell cycle stages [41,42]. Moreover, they act as scaffolds to recruit cytokinesis factors to the site of cell division and regulate the constriction of the contractile actomyosin ring [43]. In budding yeast, septins were the first substrates reported to undergo sumoylation [44,45], and in humans, but not in yeast, the sumoylation of septins is critical for septin filament bundling and cytokinesis [40].

Our data also show that the damage generated by these novel mutants of CST is sensed by the two major G2/M spindle checkpoints, as well as by the major G2/M DNA damage checkpoint. This study proposes the existence of (a) novel function(s) of the telomeric CST complex, the purpose of which might be to maintain genome integrity through the activation of Siz1 and modulation of cell cycle progression using the septins.

## 2. Materials and Methods

### 2.1. Yeast Strains and Media

The *Saccharomyces cerevisiae* yeast strains used in this study were derivatives of BF264-15Daub (*ade1 his2 leu2*-*3*,*112 trp1*-*1a ura3*Δ*ns*), described previously [17]. Yeast cultures were grown at the indicated temperatures in YEP (1% yeast extract, 2% bacto-peptone, 0.005% adenine, 0.005% uracile) supplemented with 2% glucose (YEPD) or in selective minimal media. All strains were made isogenic by back-crossing at least five times against our genetic background. The *siz1*::*KanMX4*, *siz2*::*KanMX4*, *cbf2*-*1*::*KanMX4* and *ctf13*-*30*::*KanMX4* strains were purchased from Euroscarf (Oberursel, Germany). The *bub2*Δ and *mad2*Δ mutants were kindly provided by Andrew Murray. The septin mutants were kindly provided by Michael McMurray. The *top2*-*SNM* strains and the *TOP2*-HA and *top2*-*SNM*-HA strains were kindly provided by Steve Elledge. The *rad17*Δ mutant was kindly provided by Errol Friedberg and the *mec3*Δ mutant was kindly provided by Maria-Pia Longhese. The *CDC13*-Myc_13_ strain was kindly provided by David Lydall. Constructs were prepared via polymerase chain reaction (PCR) to adapt the relevant restriction sites to the sequence of the gene (details of the constructs can be made available upon request).

The viability of cells previously grown in liquid was determined by performing and analyzing the so-called “drop tests” or “spot assays”. To do this, cells from exponential growth cultures were counted with a hematocytometer, and the cultures were then serially diluted by 1/10th, spotted onto YEPD (or selective medium) plates and incubated at the desired temperatures for 2–3 days before being photographed. In some cases, cells were just re-streaked onto YEPD plates and growth-evaluated by visualizing the numbers and sizes of the growing colonies.

### 2.2. Construction of the ten1-sz, stn1-sz and cdc13-sz Mutants

The *stn1*, *ten1* and *cdc13* mutants were generated via PCR mutagenesis, as described previously [17,18,46]. Briefly, *TEN1*, *STN1* and *CDC13* ORF flanked by sequences upstream of the ATG and post-STOP sequences were amplified via PCR (Dream Taq DNA polymerase, Thermo Fisher Scientific, Fermentas, Waltham, MA, USA) under mutagenic conditions in four distinct reactions, each reaction containing a normal concentration of one of the four dNTPs, 0.2 mM, together with imbalanced and increased concentrations of the other three dNTPs (0.5 or 1.0 mM), as well as 3.0 mM MgCl_2_ (instead of 1.5 mM) and 0.5 mM MnCl_2_, in a standard PCR buffer. Following a 30-cycle amplification, the PCR products were cleaned and, according to the gap repair method, were transformed into a double-mutant strain harboring both *siz1*Δ (::*KanMX4*) and one of *stn1*::*TRP1*, *ten1*::*LEU2* or *cdc13*::*TRP1*, each surviving, respectively, owing to a *STN1*-*URA3*, *TEN1*-*URA3* or *CDC13*-*URA3* construct, together with a *STN1*-CEN-*LEU2*, *TEN1*-CEN-*TRP1* or *CDC13*-CEN-*LEU2* plasmid (CEN for centromeric), respectively, carrying flanking regions at each extremity previously cut at unique sites at (or close to) the ATG and stop codons to linearize the plasmids and allow for recombination with the mutagenized PCR products. After the *LEU2^+^* or *TRP1*^+^ transformants plated out onto leucine- or tryptophan-lacking medium had developed at 24 °C, they were replica-plated twice onto a 5-FOA-containing medium at 24 °C to force the loss of the *STN1*-*URA3*, *TEN1*-*URA3* or *CDC13*-*URA3* plasmid. Growing colonies were then pooled together and plated out onto YEPD plates at 24 °C at various dilutions and, after colonies had developed, were replica-plated at 34 or 36 °C. After comparing them with the 24 °C master plate, colonies that failed to grow at the restrictive temperature were picked out and further expanded in a liquid culture to perform plasmid recovery (from yeast to bacteria). The recovered plasmids were transformed back into the original mutant strains to confirm their temperature sensitivity. Further details concerning the mutagenic PCR processes are available upon request.

### 2.3. FACS Analyses

To analyze the DNA content via flow cytometry, cells were fixed overnight at 4 °C in 70% ethanol, treated with RNase (1 mg/mL) and pepsin (5 mg/mL), stained with propidium iodide (50 μg/mL) and analyzed in an Attune NxT Life Technologies (Carlsbad, CA, USA) acoustic focusing cytometer.

## 3. Results

### 3.1. Overexpression of SIZ1 or TOP2 Rescues Mutants of the Telomeric Cdc13-Stn1-Ten1 Complex

All three *Saccharomyces cerevisiae* CST genes, *CDC13*, *STN1* and *TEN1*, are essential genes. In this study, we set out to generate novel mutants of the CST complex with the objective of uncovering novel functions of this complex. Because numerous genetic screens have already been performed using mutants of *S*. *cerevisiae* CST, which may somewhat tend toward saturation, we used a different and unconventional strategy. A *cdc13*Δ *stn1*Δ *ten1*Δ triple mutant expressing the wild-type *CDC13*-*TEN1*-*STN1* fusion gene from a centromeric plasmid (see Supplementary Materials, Sections S1.1 and S2.1 and Figure S1A) was perfectly viable, thereby highlighting the functionality of the *CDC13*-*TEN1*-*STN1* synthetic gene, referred to as the *CST1* gene.

The *cdc13*Δ *stn1*Δ *ten1*Δ *p*-*CDC13*-*TEN1*-*STN1* strain was then used to construct temperature-sensitive mutants (see Supplementary Materials and Figure S1A). These so-called *cdc13*Δ *stn1*Δ *ten1*Δ *cst1* mutants (referred to as *cst*Δ *p*-*cst1*, to indicate the chromosomal mutation, followed by the covering plasmid), namely *cst*Δ *p*-*cst1*-*1*, *cst*Δ *p*-*cst1*-*4*, *cst*Δ *p*-*cst1*-*5* and *cst*Δ *p*-*cst1*-*15* (Figure S1B), were then used in genetic screens to isolate suppressors of their temperature sensitivity (see Supplementary Materials, Sections S1.2 and S2.2 and Figure S2). *SIZ1*, which codes for a SUMO E3 ligase [47,48], and *TOP2*, which codes for topoisomerase II (which, interestingly, is sumoylated by Siz1, as well as by Siz2, a related SUMO E3 ligase [49]), were isolated as extragenic suppressors of these *cst*Δ *p*-*cst1* mutants (Figure S2). In these types of experiments, a rescue phenotype means an improved growth capacity and increased survival. Previously, a genome-wide screen reported the existence of negative genetic interactions between the *stn1*-*13* and the *siz1*Δ mutants [50].

Interestingly, in the same screens, *TEN1* was frequently found to rescue all four *cst*Δ *p*-*cst1* mutants (a total of 12 genomic fragments isolated; Figure S2B), but *CDC13* and *STN1* were never isolated. The overexpression of *CDC13* or *STN1* alone from a similar multicopy plasmid did not rescue the *cst*Δ *p*-*cst1* mutants, thus suggesting that the failure to isolate either one of these genes in the genetic screens was not because those two genes were missing from the libraries. Furthermore, in separate projects, genomic fragments from both libraries containing either *STN1* or *CDC13* have been frequently isolated.

### 3.2. Generation of ten1-sz, stn1-sz and cdc13-sz Mutants Rescued via SIZ1 Overexpression

In addition to the *cst*Δ *p*-*cst1* mutants rescued via *SIZ1* overexpression, it was important to also isolate single mutants for each of the three CST subunits to confirm that the effects observed were not specific to the triple-fusion protein and to simplify further studies by using more classical single-gene mutants. Of note, many previously described mutants of CST, such as *ten1*-*31*, *ten1*-*16*, *stn1*-*13*, *stn1*-*101*, *stn1*-*154*, and *cdc13*-*1* (see references [15,17,18,51]), were not rescued via *SIZ1* overexpression. Therefore, it was vital to design a strategy to isolate individual mutants for each of the three CST genes to elucidate these potentially new CST functions.

**Figure 1:**
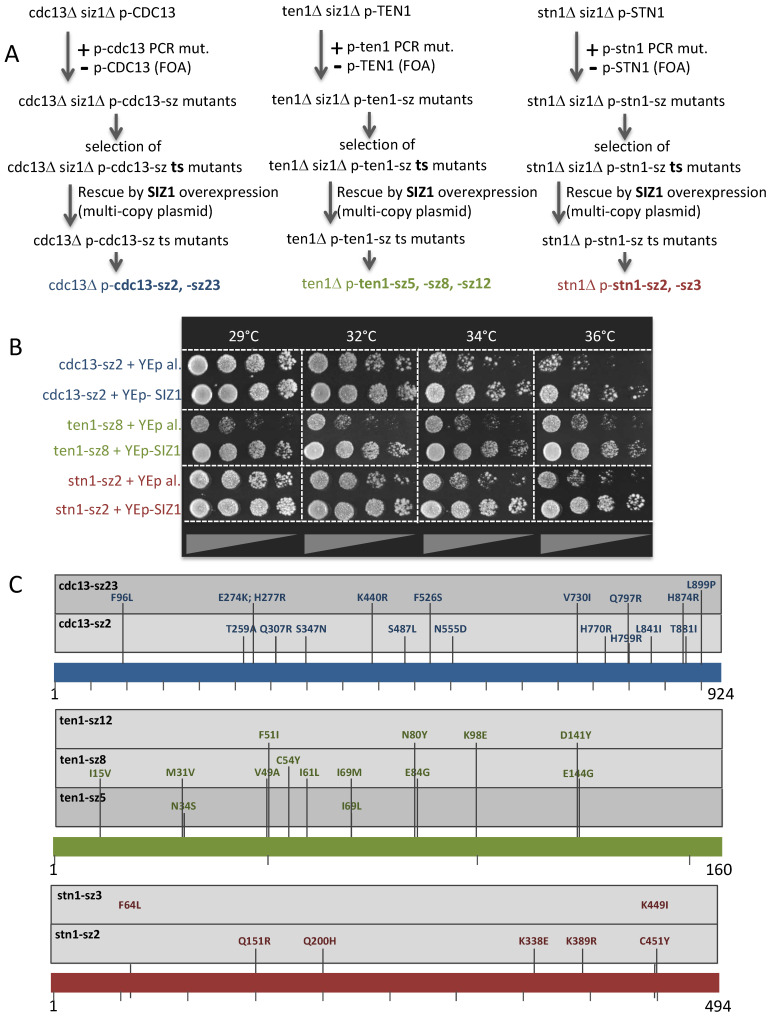
Isolation and characterization of *cdc13*-*sz*, *stn1*-*sz*, and *ten1*-*sz* mutants sensitive to intracellular Siz1 dosage. (**A**) Schematic protocol used to generate temperature-sensitive *cdc13*, *ten1*, and *stn1* mutants in a *siz1* null background (siz1Δ) via PCR mutagenesis coupled to gap repair (see Section 2.2), followed by selection of mutants that can be rescued via overexpression of *SIZ1* from a 2μ multi-copy plasmid. Two *cdc13*Δ *p*-*cdc13*-*sz* mutants (hereafter referred to as *cdc13*-*sz2* and *cdc13*-*sz23*), three *ten*1Δ *p*-*ten1*-*sz* mutants (*ten1*-*sz5*, *ten1*-*sz8*, and *ten1*-*sz12*), and two *stn1*Δ *p*-*stn1*-*sz* mutants (*stn1*-*sz2* and *stn1*-*sz3*) were isolated. (**B**) Rescue of the temperature-sensitive growth defects of the *cdc13*-*sz2*, *ten1*-*sz8*, and *stn1*-*sz2* mutants upon overexpression of *SIZ1* from a 2μ multi-copy vector (+YEp-SIZ1), with expression of plasmid alone (+YEp al.) as a control. (**C**) Sequences of all Cdc13-sz, Ten1-sz and Stn1-sz mutant proteins.

We thus generated (see Section 2.2) temperature-sensitive mutants of *CDC13*, *STN1*, and *TEN1* in a *SIZ1*-deleted background, reasoning that this might increase the chances to generate mutants more specifically defective in Siz1-related functions; the hypothesis was that a *ten1 siz1*Δ, a *stn1 siz1*Δ or a *cdc13 siz1*Δ strain would be more prone to generate temperature-sensitive clones than the corresponding *SIZ1*^+^ strains, and that these would be more specifically defective in Siz1-related functions (Figure 1A, top panels). In fact, this is exactly what happened. Indeed, in the case of *stn1 siz1*Δ, only three temperature-sensitive clones (named *stn1*-*sz* mutants, *sz* referring to Siz1 involvement in *stn1* phenotypes) were isolated from approximately 20,000 transformants. The *stn1*-*sz1* no longer rescued the original strain upon re-transformation, but *stn1*-*sz2* and *stn1*-*sz3* passed this test. Interestingly, the temperature sensitivity of both, higher for *stn1*-*sz2* than for *stn1*-*sz3*, could be rescued by overexpressing *SIZ1*, clearly visible on the increased capacity to form colonies, as well as on the partial suppression of morphological defects (Figure 1B for *stn1*-*sz2).* Using the same approach, two mutants of *CDC13* (*cdc13*-*sz2* and *cdc13*-*sz23*), selected among thirty-three temperature-sensitive clones isolated from around 5000 transformants, could be rescued via *SIZ1* overexpression. Similarly, the temperature sensitivities of three *TEN1* mutants (*ten1*-*sz5*, *ten1*-*sz8* and *ten1*-*sz12*) selected among thirteen temperature-sensitive clones in a total of around 10,000 transformants were partially suppressed at 34 °C and 36 °C upon the overexpression of *SIZ1* (Figure 1B). The sequences of all the *cst*-*sz* mutants are provided in Figure 1C.

All of the *cst*-*sz* mutants tested, namely *stn1*-*sz2 siz1*Δ, *cdc13*-*sz2 siz1*Δ, *cdc13*-*sz23 siz*1Δ, and *ten1*-*sz8 siz1*Δ, grew no worse than their *SIZ1*^+^ counterparts, except perhaps the *ten1*-*sz8 siz1*Δ mutant. Nevertheless, the overexpression of *SIZ1* clearly rescued these mutants, pointing to the involvement of Siz1 in their loss of function. Therefore, these *cdc13*-*sz*, *stn1*-*sz* and *ten1*-*sz* mutants appear to be sensitive to intracellular Siz1 dosage.

### 3.3. All Three Major Mitotic Checkpoints Detect Damage in the stn1-sz2 Mutant

**Figure 2:**
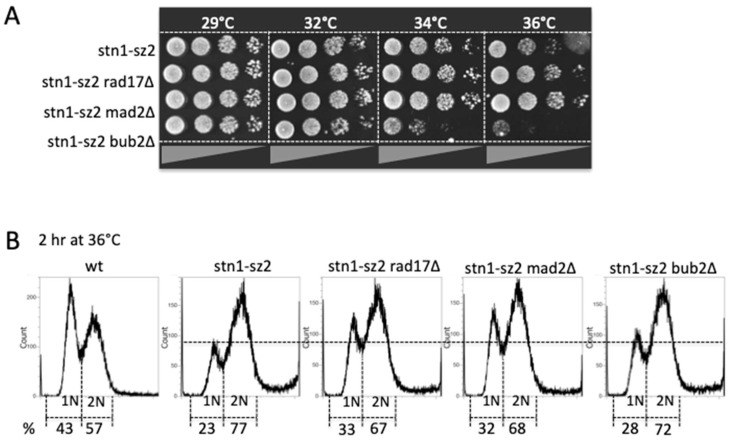
Effects on the temperature-sensitive *stn1*-*sz2* mutant of genetic inactivation of the G2/M DNA damage checkpoint (*rad17*Δ mutation) or of one the two spindle checkpoints (*mad2*Δ and *bub2*Δ mutations) on cell viability (**A**) and on cell cycle distribution assessed using FACS analysis (**B**).

Our next objective was to determine whether or not the damage in the *cst1*-*sz* mutants was activating one or more of the three major G2/M checkpoints, namely the Mec1-Mec3 DNA damage checkpoint [52], the spindle orientation checkpoint [53] and the spindle assembly checkpoint [54]. All three major G2/M checkpoints played a role in the detection of *stn1*-*sz2* defects, as attested by the visible effects on viability when the DNA damage checkpoint or one of the two spindle checkpoints had been genetically inactivated in the *rad17*Δ, *mad2*Δ and *bub2*Δ mutants, respectively (Figure 2A). Interestingly, two different effects of these checkpoint mutations on *stn1*-*sz2* growth were observed. Thus, the inactivation of *RAD17* or of *MAD2* in the *stn1*-*sz2* mutant at 34–36 °C resulted in an increase in cell viability, indicated by the improvement of colony formation or colony size (Figure 2A). This phenotype was reminiscent of that described for the *cdc13*-*1 rad17*Δ mutant and is, in fact, characteristic of forcing the passage of the checkpoint-mediated cell cycle arrest [55,56]. This same phenotype has also been described for the *cdc13*-*1 bub2*Δ mutant [57] and has been interpreted in the same way as that of the *cdc13*-*1 rad17*Δ mutant [55,56]. On the other hand, the phenotype of the *stn1*-*sz2 bub2*Δ double mutant was more classical, exhibiting a strong synthetic lethality indicative of the dramatic consequences of preventing the activation of the Bub2 checkpoint in the presence of *stn1*-*sz2* damage (Figure 2A).

A DNA content analysis via flow cytometry (FACS) confirmed the involvement of the Mec1-dependent DNA damage and Mad2-dependent spindle checkpoints in the arrest of the *stn1*-*sz2* mutant at the restrictive temperature of 36 °C (Figure 2B). On the other hand, the *stn1*-*sz2 bub2*Δ mutant cells were distributed along the different cell cycle stages just like the *stn1*-*sz2* cells, thus confirming that synthetic lethality rather than the deregulation of cell cycle arrest was taking place. In summary, the *stn1*-*sz2* mutant at 36 °C produces damage that normally triggers cell cycle arrest at G2/M, and we observe that the complete genetic inactivation of either *RAD17* or *MAD2* prevents that arrest, while the inactivation of *BUB2* in *stn1*-*sz2* kills the cells. Thus, all three G2/M checkpoint pathways detected damage produced in the *stn1*-*sz2* mutant.

### 3.4. Sumoylation of Top2 and Septins in the Mutants of CST

As seen above, sumoylation appears to play some role in the rescue of the CST mutants described in this study. At least two likely candidates emerged as being possibly involved in this: the septins and Top2.

We found that Top2 was sumoylated in the *stn1*-*sz2* mutant (Section S2.3 and Figure S3A), and that Cdc3 septin was also sumoylated in this mutant, as well as in the *ten1*-*sz* and *cdc13*-*sz* mutants (Figures S3B,C and S4). However, in neither case could we establish whether the sumoylation of Top2 and Cdc3 resulted from the damage inflicted by the mutations in CST, or whether it was rather due to the fact that mutant cells were arrested in mitosis as a consequence of the cell cycle effect of the temperature-sensitive mutations.

**Figure 3:**
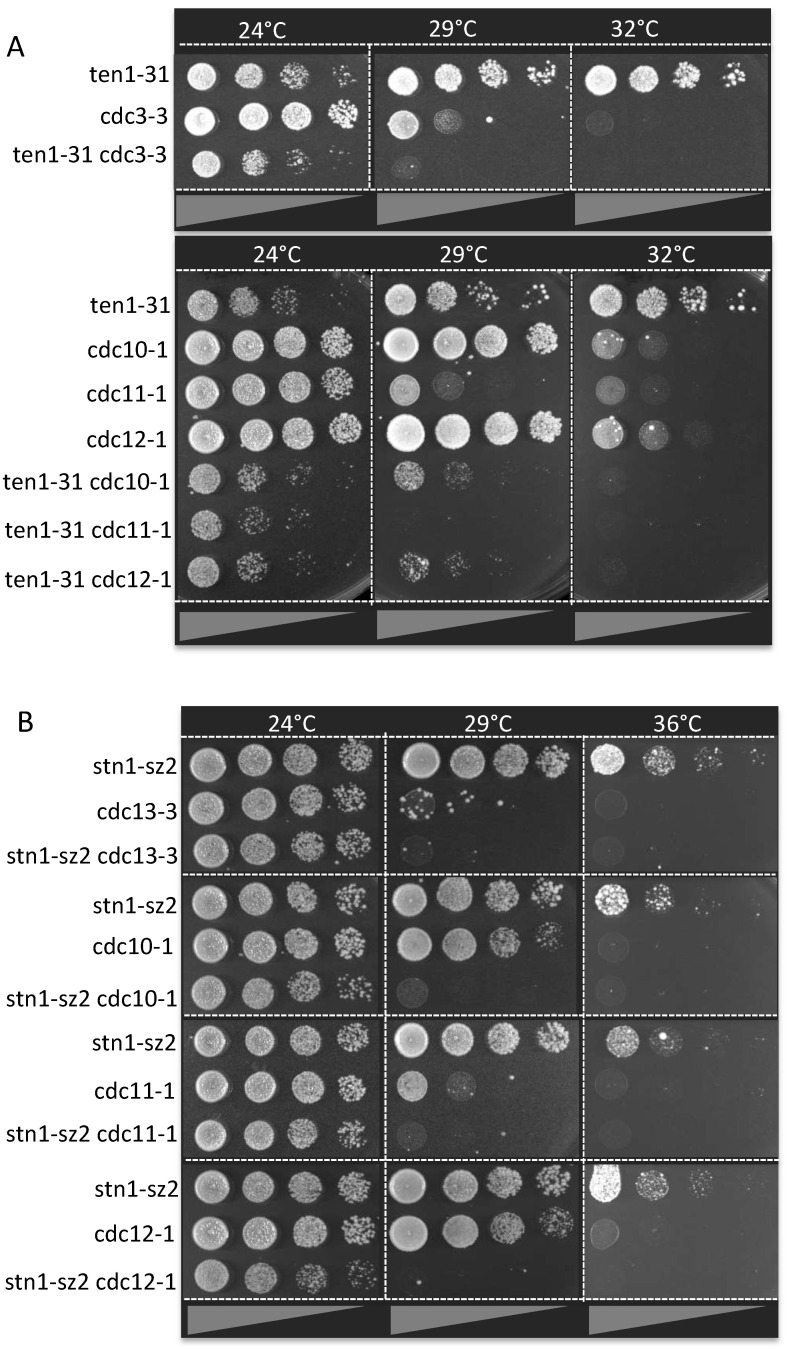
Genetic interactions between the Cdc13-Stn1-Ten1 complex and septins. (**A**) The temperature-sensitive *ten1*-*31* mutant exhibited negative genetic interactions (also frequently referred to as synthetic growth defects) with the indicated temperature-sensitive septin mutants. (**B**) The temperature-sensitive *stn1*-*sz2* mutant exhibited strong genetic interactions with several temperature-sensitive mutants of septin, as indicated.

Previous studies have established septins as major targets of Siz1 SUMO ligase activity [47,48,58,59]. Among the five *S*. *cerevisiae* septins functioning during vegetative growth, Cdc3 is the principal target of Siz1-dependent sumoylation (four lysine residues were identified), followed by Shs1 (two lysine residues) and Cdc11 (one lysine residue) [48,58]. In a SUMO interactome study, all five septins (Cdc3, Cdc10, Cdc11, Cdc12 and Shs1) were found to bind Siz1 [60]. In addition, in a proteome-wide study, Cdc12, as well as Cdc3, Cdc11 and Shs1, were also found to be sumoylated [61]. Interestingly, negative genetic interactions between *TEN1* and *CDC12* were uncovered in a previous study [62]. In our study, we found that cell viability (Figure 3A) and morphological defects were much aggravated when the *ten1*-*31* mutation and a mutation in a septin gene were combined, compared with the single mutants alone (Figure 3A). This contrasted with two well-documented mutant alleles of *CDC13* and *STN1*, *cdc13*-*1* [15] and *stn1*-*13* [17], which exhibited moderate genetic interactions with a *cdc10* (but not *cdc11* or *cdc12*) and a *cdc12* (but not *cdc10* or *cdc11*) mutant, respectively. Importantly, stronger negative genetic interactions were observed between the septin mutants and the *stn1*-*sz2* mutant (Figure 3B) than between the septin mutants and the classical *stn1*-*13* and *cdc13*-*1* mutants. These new *cst1*-*sz* mutants thus have a loss of function that is more sensitive to the presence of intact septins than the mutants of CST already described in the literature.

**Figure 4:**
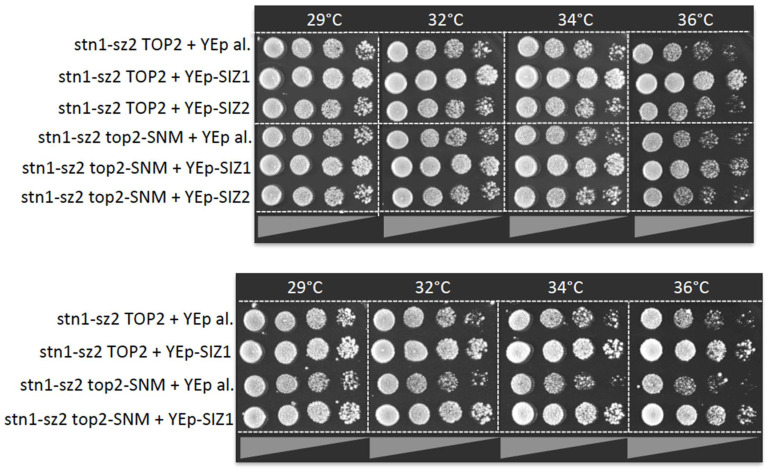
Effects of Top2 sumoylation on the rescue of the *stn1*-*sz2* mutant. The rescue of this mutant via overexpression of *SIZ1* (or of *SIZ2* or of vector alone; all from a 2μ multi-copy plasmid, “YEp-SIZ1”, “YEp-SIZ2”, or “YEp al.”) was slightly less efficient when *top2*-*SNM* mutations were expressed from *TOP2* locus, rendering Top2 unsumoylatable. Top and bottom panels represent two independent experiments.

The sumoylated form of Top2 represents a metaphase–anaphase checkpoint both in yeast and humans [63], and Siz1 is predominantly responsible for Top2 sumoylation [49]. To check whether the sumoylation of Top2 was involved in the rescue of the CST loss of function via *SIZ1*, we used a mutant of *TOP2*, *top2*-*SNM* (Sumo No More), which encodes a protein with six mutations in lysine residues sumoylated using SUMO/Smt3 [64]. In some experiments, the overexpression of *SIZ1* was just a little more efficient in rescuing *stn1*-*sz2* than in rescuing the isogenic *stn1*-*sz2 top2*-*SNM* strain (Figure 4, top panels), but this was not the case in all experiments (Figure 4, bottom panels). These experiments therefore suggest that Top2 sumoylation might not play a determinant role in these mechanisms of rescuing *stn1*-*sz2* via *SIZ1* overexpression.

## 4. Discussion

In this study, we isolated novel mutants of the conserved telomeric Cdc13-Stn1-Ten1 (CST) complex of the yeast *Saccharomyces cerevisiae*. These mutants are unique in the way the damage they generate simultaneously activates both major G2/M spindle checkpoints together with the G2/M DNA damage checkpoint. In cancer research, a significant challenge is to accurately identify the origin and nature of the damage generated at the telomeres and the way this is recognized by the checkpoint machineries, which in turn will affect the cell cycle progression in the tumor cells. Understanding the mechanisms by which telomeric damage controls the cell cycle is thus of major importance in cancer biology, as well as in aging-related (and other) telomeropathies [6,8,65]. The conserved telomeric CST (CTC1-STN1-TEN1) complex plays major roles in these pathways, particularly by providing telomere end protection and the regulation of telomerase accessibility [10,11,27,31,66].

### 4.1. CST Mutants Rescued via Overexpression of SIZ1 or TOP2

The new mutants of CST isolated here represent invaluable tools to study (a) yet uncharacterized function(s) of CST. The unconventional strategy of genetic screening designed here to isolate these CST mutants presumably introduced a bias towards the isolation of this particular class of CST mutants. A particular unnatural conformation adopted by the mutagenized *cdc13*-*ten1*-*stn1* hybrid gene (we call it the *CST1* gene) might have favored the accumulation of unusual mutations.

This study’s main conclusion is that defects in *S*. *cerevisiae* CST are simultaneously sensed not only by the G2/M DNA damage checkpoint, but also by the two major G2/M spindle checkpoints. Most of the mutants of CST identified to date activate the Mec1-mediated DNA damage checkpoint [52], due to the fact that most of them are compromised in telomeric DNA binding and accumulate abnormal levels of single-stranded telomeric DNA in budding yeast [15,46] and other organisms [11,31,67]. The Bub2-dependent checkpoint monitors correct the orientation of the mitotic spindle [53], while the Mad2-dependent checkpoint monitors the attachment of the kinetochores (centromeric structures of the chromosomes) to the spindle microtubules [54]. It should be noted that the *cdc13*-*1* mutant has previously been reported to activate the Bub2 spindle orientation checkpoint [57]. While the pathways at the origin of this genetic interaction have not yet been elucidated, it is tempting to speculate that they might correspond to those suspected to be deregulated in our new mutants of CST.

In addition to *SIZ1* and *TOP2*, *TEN1* (but not *STN1* and *CDC13*) was also a good suppressor of the *cst*Δ *p*-*cst1* mutants, perhaps highlighting the fact that the essential domains of Ten1 are masked by the surrounding Cdc13 and Stn1 in the synthetic fusion protein. In this respect, it would be interesting to construct temperature-sensitive hybrid fusion CST genes with other orders of arrangement of the three genes within the fusion to see whether their subsequent mutagenesis also leads to isolation of *SIZ1* and *TOP2* extragenic suppressors. In addition, performing structural modeling of the fusion encoded by the synthetic *CST1* gene might allow us to pinpoint the particular losses of interactions with other mitotic and/or telomeric actors when compared with structural data from each of the single CST subunits.

Interestingly, mutagenesis of each of the single CST genes under conditions of complete depletion of intracellular Siz1 (in a *siz1*Δ background), also introduced a bias in the isolation of temperature-sensitive mutants that could be rescued via the overexpression of *SIZ1*. Indeed, under such conditions (*siz1*Δ background), it became much easier to isolate temperature-sensitive alleles than under normal conditions, as shown in our genetic screenings. These so-called *cdc13*-*sz*, *stn1*-*sz* and *ten1*-*sz* mutants might be more amenable to future structural and genetic studies, as the characteristics of the mutant proteins might be directly compared to those of the corresponding wild-type proteins.

Another finding of this study is to have generated a novel, artificial, fusion hybrid gene that is fully functional in terms of complementing the *cdc13*Δ *stn1*Δ *ten1*Δ triple-deletion mutant. We chose to name this synthetic gene *CST1* rather than *CTS1* (which would have been more logical given the order of the genes in the fusion) because *CTS1* is an already deposited gene name (encoding endochitinase) and also because CST is the name of the complex composed of Cdc13, Stn1, and Ten1, and the name *cst1* for these mutants is therefore more descriptive of their functions. This functional synthetic *CST1* gene will be a useful tool in future studies aiming to further decipher the structural interactions of the three subunits with other telomeric proteins.

### 4.2. Mechanisms of Rescue of Mutants of CST via Overexpression of SIZ1 or TOP2

The isolation of *SIZ1* and *TOP2* as extragenic multicopy suppressors of the newly described *cst1* mutants led us to examine the possibility that SUMO post-translational modifications [44,68,69,70] might be involved in these mechanisms of rescue. We found here that the rescue of CST mutants via the overexpression of *TOP2* did not strongly depend on Top2 sumoylation, an event that is essential for a metaphase–anaphase checkpoint both in yeast and humans [63]. Several additional arguments suggested that the sumoylation of the septins was also possibly involved in the mechanisms of the rescue of the new *cst1* mutants via the overexpression of *SIZ1*, but this remains to be demonstrated.

Given the diversity of mechanisms of the extragenic rescue of mutations described in the literature (see Discussion in Supplementary Materials, Section S3.1), future studies will be needed for a complete understanding of how exactly the overexpression of *SIZ1* or of *TOP2* leads to diminished damage and increased viability in these new mutants of CST.

Since the new mutants of *S*. *cerevisiae* CST described here activate the two major spindle checkpoints, the physical association of hCST with cohesin [29] might be pertinent to the mechanisms studied here in yeast. In addition, we need to understand how septin sumoylation can affect the cell cycle progression in response to damage, with studying such mechanisms becoming pertinent following the recent finding that a defect in septin sumoylation in humans correlates with defects in cytokinesis [71].

### 4.3. Conclusions

This study’s main conclusion is that defects in *S*. *cerevisiae* CST (Cdc13-Stn1-Ten1) are sensed not only by the G2/M DNA damage checkpoint, but also by the two major G2/M spindle checkpoints. The pathologies associated with mutations in human CTC1 and STN1, mainly dyskeratosis congenita and Coats Plus ([72] and references therein), might therefore stem not only from deregulation in CTC1-STN1-Pol-α-telomerase interactions [73], but also from potential defects in mitotic spindle stability conferred by the mutations in *S*. *cerevisiae* CST described here. Our data suggest that septins might represent a potential target for the spindle checkpoints that become activated after telomeric damage in these new mutants of CST.

## Data Availability

All the data supporting the reported results are present in this article (main text and Supplementary Materials). Additional data or protocol details are available upon request.

## Supplementary Materials

The following supporting information can be downloaded at: https://www.mdpi.com/article/10.3390/cells13191605/s1, Figure S1: Construction and characterization of *cdc13*Δ *stn1*Δ *ten1*Δ mutants expressing temperature-sensitive *cdc13*-*ten1*-*stn1* fusion mutant genes. Figure S2. *SIZ1* and *TOP2* rescue mutants of the Cdc13-Stn1-Ten1 complex. Figure S3. Sumoylation of Top2-HA_2_ and septin sumoylation. Figure S4. HA_2_-Cdc3 sumoylation peaked at G2/M and stayed very low during the rest of the cell cycle. References [74,75,76,77,78,79,80,81,82] are cited in the supplementary materials.
